# A role for Mfb1p in region-specific anchorage of high-functioning mitochondria and lifespan in *Saccharomyces cerevisiae*

**DOI:** 10.1038/ncomms10595

**Published:** 2016-02-03

**Authors:** Wolfgang M. Pernice, Jason D. Vevea, Liza A. Pon

**Affiliations:** 1 Department of Pathology and Cell Biology, Columbia University, 630 West 168th Street, P&S 14-442, New York, New York 10032, USA

## Abstract

Previous studies indicate that replicative lifespan in daughter cells of *Sacchraromyces cerevisiae* depends on the preferential inheritance of young, high-functioning mitochondria. We report here that mitochondria are functionally segregated even within single mother cells in *S. cerevisiae*. A high-functioning population of mitochondria accumulates at the tip of the mother cell distal to the bud. We find that the mitochondrial F-box protein (Mfb1p) localizes to mitochondria in the mother tip and is required for mitochondrial anchorage at that site, independent of the previously identified anchorage protein Num1p. Deletion of *MFB1* results in loss of the mother-tip-localized mitochondrial population, defects in mitochondrial function and premature replicative ageing. Inhibiting mitochondrial inheritance to buds, by deletion of *MMR1*, in *mfb1Δ* cells restores mitochondrial distribution, promotes mitochondrial function and extends replicative lifespan. Our results identify a mechanism that retains a reservoir of high-functioning mitochondria in mother cells and thereby preserves maternal reproductive capacity.

## Abstract (web summary)

Mitochondria are asymmetrically inherited during cell division, a process that can affect cell fate and lifespan. Here the authors describe a mechanism for mitochondrial quality control in yeast that maintains a reservoir of high-functioning mitochondria in mother cells and preserves maternal reproductive capacity.

## Graphical abstract

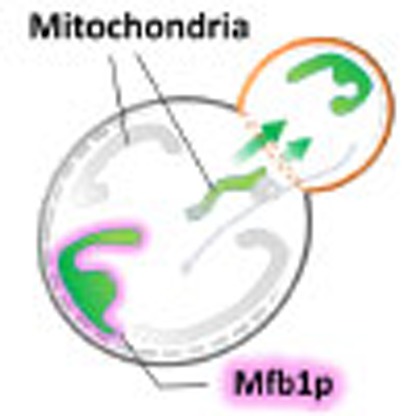

A hallmark of ageing is the decline of mitochondrial function^1^,^2^. Activating pathways of mitochondrial quality control delays ageing and extends lifespan in multiple organisms^3^,^4^,^5^. However, the homeostatic processes of mitochondrial repair and regeneration eventually fail with age and mitochondrial function declines^6^.

In *S. cerevisiae*, mother cells produce a limited number of daughters over their lifespan and continue to age with each bud produced; however, daughter cells are born young, largely independent of the age of their mother cells. This finding, that yeast undergo mother–daughter age asymmetry, led to the model that ageing determinants are retained in mother cells, while rejuvenating factors are selectively inherited by buds. Indeed, ageing determinants including oxidatively damaged protein aggregates and extrachromosomal rDNA circles are selectively retained in mother cells. In contrast, antioxidant activity is increased in daughter but not in mother cells after cytokinesis. Daughter cells also inherit more acidic vacuoles compared with vacuoles in mother cells. Finally, defects in the asymmetric inheritance of these ageing or rejuvenating factors results in defect in lifespan control^4^,^7^,^8^,^9^.

Yeast daughter cells also preferentially inherit fitter mitochondria, which are more reducing and harbour lower levels of reactive oxygen species (ROS) compared with mitochondria in mother cells. Defects in the asymmetric inheritance of mitochondria also compromise mother–daughter age asymmetry^10^. Recent studies indicate that mitochondria are also asymmetrically inherited in mammalian cells, and that this process affects cell fate. Specifically, self-renewal of mammary stem-like cells depends on the preferential inheritance of young mitochondria, while older organelles segregate with somatic cell fates^11^.

The mechanism underlying asymmetric inheritance of mitochondria in mammalian cells is not well understood. However, two mechanisms have been identified that promote inheritance of fitter mitochondria by yeast daughter cells. In one mechanism, the cytoskeleton drives transport of higher-functioning mitochondria from mother cells to buds and transport of low-functioning mitochondria in the opposite direction, from buds to mother cells. In the other, acidification of the vacuole maintains mitochondrial membrane potential in buds but not in mother cells. Manipulating either of these mitochondrial quality-control pathways to reduce or increase mitochondrial quality in daughter cells results in shorter or longer replicative lifespans (RLSs), respectively^4^,^5^.

Although the mechanisms that drive the preferential partitioning of fitter mitochondria to daughter cells are a topic of active investigation, it is unclear whether and how high-quality mitochondria must also be retained within the mother cell. Our previous studies revealed accumulation of mitochondria within the tip of the mother cell distal to the bud, but not the mechanisms or consequences of this process^12^. Other studies indicate that mitochondrial F-box protein (Mfb1p) is required for normal mitochondrial morphology. Mfb1p is a non-canonical F-box protein that interacts with Skp1p but not the larger Skp1-Cullin-F-box ubiquitin ligase complex or the proteasome and is required for normal mitochondrial distribution. Mfb1p has the capacity to bind to mitochondria. Moreover, it is asymmetrically distributed within yeast. Specifically, it is enriched on mitochondria in yeast mother cells^12^,^13^. We show here that Mfb1p is required to retain a reservoir of high-quality mitochondria at the tip of dividing mother cells distal to the bud and for normal mother cell RLS.

## Results

### High-functioning mitochondria are anchored in mother tips

**Figure 1:**
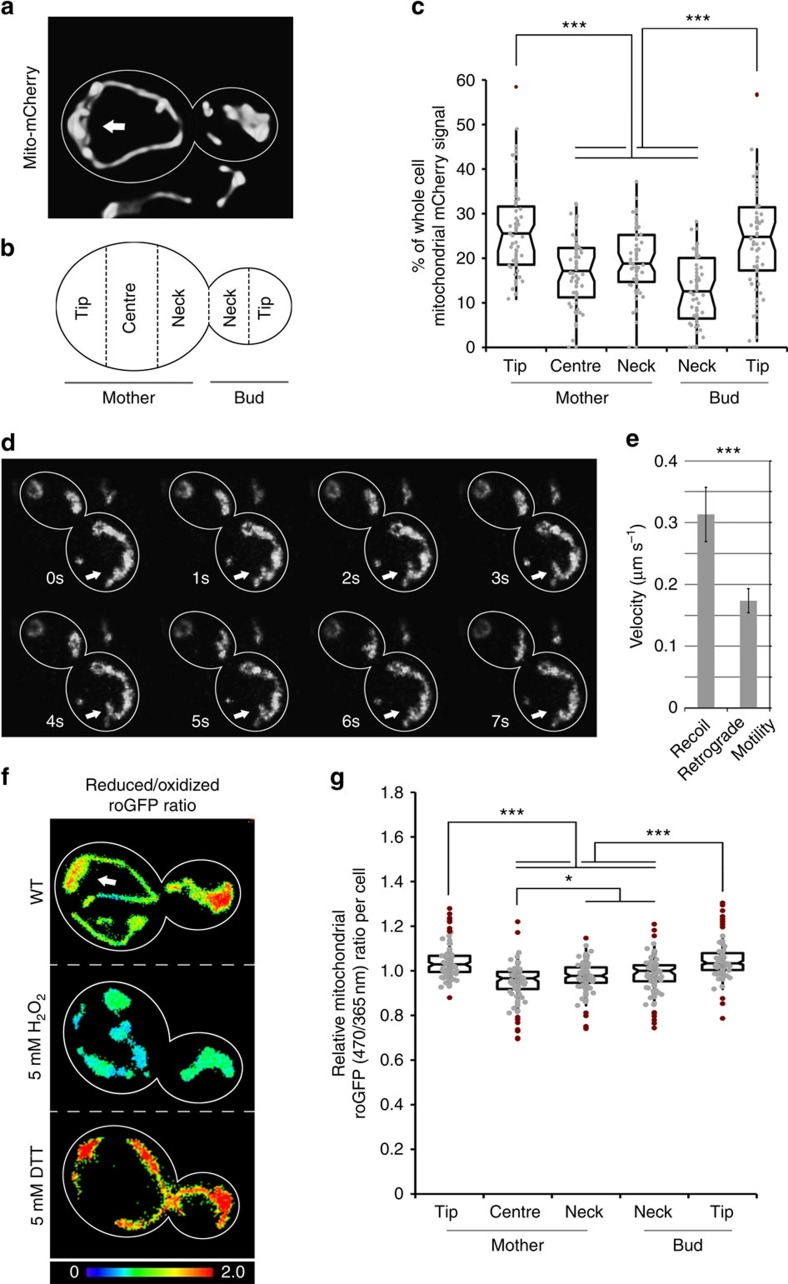
A population of higher-functioning mitochondria are anchored to and accumulate at the tip of the mother cell. (**a**–**c**) Wild-type yeast cells that express the mitochondria-targeted fusion protein, Cit1p-mCherry, were grown to mid-log phase and visualized by fluorescence microscopy. (**a**) Representative volume rendering of mitochondria. Cell outlines are shown in white. Arrow points to mitochondria accumulated in the mother cell tip. (**b**) Schematic indicating regions of interest for quantification of mitochondrial distribution. (**c**) Notched box plot of mitochondrial distribution in cells. Mitochondrial distribution was determined by measuring the integrated fluorescence of Cit1p-mCherry in regions defined in **b**. The central band in the box represents the median, boxes indicate the middle quartiles and whiskers extend to the 5th and 95th percentiles; red dots indicate data points beyond this range. Data are representative of three independent experiments (*n*=40). (**d**) Frames from a time-lapse series showing a mitochondrion (arrows) that undergoes bud-directed movement from the mother cell tip and then rapidly recoils to the mother tip. (**e**) Velocity of mitochondria undergoing mitochondrial ‘springback' events, defined as described in Methods, and typical retrograde motility. Error bars represent s.e.m. (**f**) Wild-type (WT) cells expressing mito-roGFP1 were grown to mid-log phase and visualized by fluorescence microscopy. Images are reduced/oxidized mito-roGFP1 ratios. Colour scale indicates ratio values; higher numbers and warmer colours indicate more reducing mitochondria. (**g**) Quantification of the relative mitochondrial roGFP1 ratio as a function of position within WT yeast cells containing large buds (bud diameter>50% of mother diameter). Local ratios were normalized to the ratio for the cell as a whole. *n*>40 cells. Data are representative of three trials. Scale bars, 1 μm. Statistical significance was determined by Student's *t*-test. *P<0.05, **P<0.01 and ***P<0.005.

Our previous studies revealed that mitochondria accumulate in and are anchored at the bud tip in budding yeast, and that those mitochondria are higher functioning compared with mitochondria in mother cells^7^,^14^. Here we describe the mother cell tip as a second region for accumulation and anchorage of higher-functioning mitochondria. First, using a fluorescently tagged mitochondrial matrix protein (Cit1p-mCherry) to visualize mitochondria in living yeast cells, we confirmed our previous lower-resolution observations^15^ that mitochondria accumulate in the mother cell tip. Quantitative analysis of the abundance of mitochondria as a function of position in the yeast cell reveals that the proportion of mitochondria localizing to the mother tip is significantly higher than for other regions of the mother cell and comparable to the enrichment of mitochondria in the bud tip (Fig. 1a–c).

Next, we tested whether mitochondria that accumulate in the distal tip of yeast mother cells are anchored at that site. Here we reasoned that mitochondria that are anchored in the mother cell tip may also interact with the anterograde motility apparatus. If so, they will be under tension, and if the forces for anchorage of mitochondria in the mother cell tip are greater than the forces for bud-directed anterograde movement, then mitochondria that move away from the mother cell tip will recoil back to the anchorage site. Indeed, analysis of mitochondrial motility using three-dimensional (3D) time-lapse imaging at high temporal resolution revealed mitochondrial ‘springback' events at the mother tip (Fig. 1d). These springback events are distinct from retrograde movement from the bud towards the mother cell: the recoil occurs with significantly higher velocity than normal retrograde mitochondrial trafficking along actin cables (Fig. 1e). Although we cannot exclude as-yet unknown mechanisms that may drive retrograde velocity during springback, we suggest that it is driven by elastic tension within an organelle anchored at one end.

Finally, our previous studies revealed that mitochondria within mother cells can be functionally distinct^7^. However, these studies did not determine whether mitochondrial function is linked to their localization within the yeast mother cell. To correlate distribution with mitochondrial quality parameters, we assessed mitochondrial redox state as a function of position within mother cells using a redox-sensitive green fluorescent protein (GFP) variant targeted to the mitochondrial matrix (mito-roGFP1). In response to reduction or oxidation of the thiols of introduced cysteine residues, mito-roGFP1 exhibits distinct excitation maxima^16^,^17^. Thus, measuring the ratio of reduced to oxidized mito-roGFP signal allows for real-time, ratiometric assessment of local redox states in mitochondria^18^.

The dynamic range of mito-roGFP can be determined by measuring the reduced to oxidized ratio of mito-roGFP1 after treatment with oxidizing or reducing agents. Treatment of yeast with the reducing agent dithiothreitol or the oxidizing agent H_2_O_2_ results in a 40% increase and 20% decrease in the mito-roGFP ratio, respectively (Fig. 1f and Supplementary Fig. 1a). Using mito-roGFP1, we confirmed that daughter cells preferentially inherit mitochondria that are more reduced and therefore higher functioning compared with mitochondria in mother cells (Fig. 1g). Beyond this, we find that mitochondria that are anchored at the mother tip of wild-type cells are 8–10% more reduced and therefore higher functioning compared with mitochondria in other maternal regions. Indeed, the redox state of mitochondria in the mother cell tip is similar to that of mitochondria that are anchored in the bud tip (Fig. 1f,g). Thus, we find that mitochondria accumulate in and are anchored at the mother cell tip, and that these mitochondria are higher functioning compared with other mitochondria in the mother cell.

### Mfb1p is required for mitochondrial retention in mother tips

**Figure 2:**
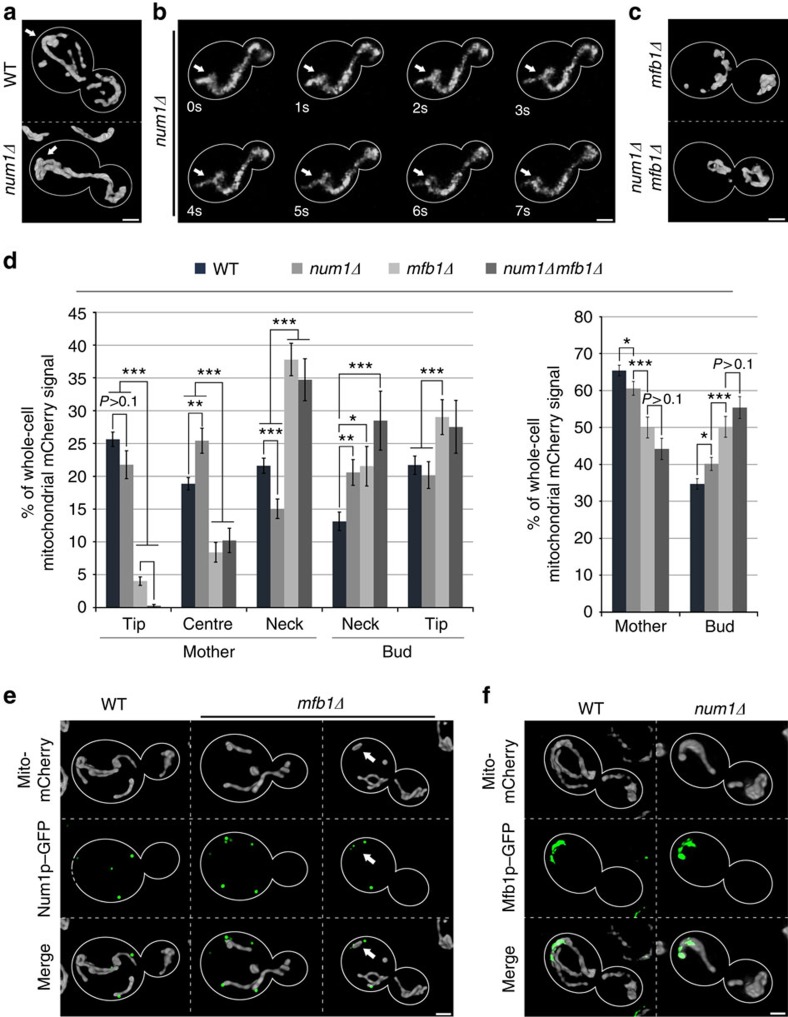
Mfb1p localizes to the mother cell tip and is required for Num1p-independent anchorage of mitochondria at that site. (**a**–**c**) Cells expressing Cit1p-mCherry were grown to mid-log phase and imaged by fluorescence microscopy. Cell outlines are shown in white. (**a**) Representative 3D renderings of mitochondria in wild-type (WT) and *num1Δ* cells. Arrows point to mitochondria that accumulate in the mother cell tip in both genotypes. (**b**) Frames from a representative time-lapse series showing a mitochondrial ‘springback' event at the mother tip of a *num1Δ* cell. Arrows mark the initial position of a mitochondrion that undergoes anterograde movement (*t*=1–6 s) and retraction (*t*=6–7 s). (**c**) Representative 3D renderings of mitochondria in in *mfb1Δ* and *num1Δ mfb1Δ* cells. (**d**) Quantification of the relative distribution of mitochondrial Cit1p-mCherry in WT, *num1Δ*, *mfb1Δ* and *num1Δ mfb1Δ* cells in five zones as defined in Fig. 1b. Error bars show the s.e.m. (*n*>40). Data are representative of three independent experiments. (**e**,**f**) Cells expressing Cit1p-mCherry and endogenously tagged Num1p-GFP or Mfb1p-GFP fusion proteins were grown to mid-log phase and imaged by fluorescence microscopy. (**e**) Representative 3D renderings of mitochondria and Num1p-GFP in WT and *mfb1Δ* cells. White arrows indicate residual Num1p-dependent mitochondrial retention at the mother tip in the absence of Mfb1p. (**f**) Representative 3D renderings of mitochondria and Mfb1p-GFP in WT and *num1Δ* cells. Scale bars, 1 μm. Statistical significance was determined using Student's *t*-test. *P<0.05, **P<0.01 and ***P<0.005.

Previous studies identified Num1p as a protein that mediates anchorage of mitochondria to the cortex of yeast mother cells^19^,^20^,^21^. We confirmed that deletion of *NUM1* abolishes mitochondrial localization to most of the maternal cortex (Fig. 2a). Interestingly, however, accumulation of mitochondria in the mother cell tip occurs even in the absence of *NUM1* (Fig. 2a,d). Deletion of *NUM1* also does not affect physical anchorage of mitochondria in the mother cell tip: mitochondria exhibit springback events at that site even in *num1Δ* cells. (Fig. 2b). Thus, there is anchorage of mitochondria in the mother cell tip that is independent of Num1p.

To identify alternative maternal mitochondrial retention factors, we studied genes that showed positive genetic interactions with Mmr1p^22^,^23^. Previous studies indicate that Mmr1p, a member of the DSL1 family of tethering proteins, is required for efficient mitochondrial inheritance by mediating anchorage of mitochondria in the bud tip and by serving as an adapter that links mitochondria to a type V myosin motor^14^,^24^,^25^,^26^. Deletion of *MMR1* causes severe defects in the accumulation of mitochondria in the bud. We expected that deletion of genes that have positive genetic interactions with *MMR1* should conversely promote accumulation of mitochondria in buds, potentially by disrupting anchorage of the organelle in the mother cell tip.

Among the strongest positive genetic interactions for *MMR1* was *MFB1* (refs 22, 23). We therefore examined mitochondrial distribution within *mfb1Δ* cells. Strikingly, deletion of *MFB1* resulted in specific depletion of mitochondria from the mother cell tip by 86% compared with wild-type cells, and a dramatic shift of mitochondrial mass towards the mother cell neck and into the daughter cell (Fig. 2c,d). This was not due to changes in mitochondrial motility (Supplementary Fig. 2a–c). Thus, the accumulation of mitochondria at the mother cell tip largely depends on Mfb1p.

Interestingly, despite the loss of mitochondrial mass from the mother tip, many cells retained at least one small mitochondrial fragment at the mother tip, suggesting that anchorage of mitochondria at this site was still not categorically abolished in *mfb1Δ* cells (Fig. 2d,e and Supplementary Fig. 2d). Therefore, we asked whether mitochondrial retention at the mother tip in the absence of Mfb1p was due to residual anchorage through Num1p. Indeed, deletion of *NUM1* in *mfb1Δ* cells fully abolished mitochondrial anchorage at the mother tip and aggravated the maternal retention defect observed in *mfb1Δ* cells (Fig. 2c,d). Together, these findings indicate that Mfb1p plays a major role in region-specific anchorage of mitochondria in the mother cell tip and Num1p plays a minor role in this process, through its function as a cortical anchor for mitochondria throughout the mother cell.

To further assess the function of Mfb1p and Num1p in retention of mitochondria in mother cells, we studied the localization of both proteins. Previous studies revealed that Mfb1p is enriched in the mother cell tip and Num1p localizes to punctate structures at sites where mitochondria are closely apposed to the mother cell cortex^12^,^19^. We confirmed this localization of Num1p (Fig. 2e). Moreover, using optical sectioning, 3D reconstruction and digital deconvolution to visualize Mfb1p in living yeast (Fig. 2f) and quantitative analysis of the abundance of Mfb1p as a function of position within yeast cells (Supplementary Fig. 5d), we find that the protein localizes to mitochondria that are anchored to the mother cell tip and is selectively enriched at that site. We also find that Mfb1p and Num1p localize independently: Mfb1p is not required for normal localization of Num1p or for normal levels of Num1p puncta at the cell cortex. Conversely, Num1p is not required for normal localization of Mfb1p (Fig. 2e,f and Supplementary Fig. 2e–g). Finally, we find that Num1p puncta localize to residual mitochondria that are anchored at the mother cell tip in *mfb1Δ* cells (Fig. 2e and Supplementary Fig. 2g). Thus, we obtained additional evidence that this population is anchored there by Num1p. Together, these findings indicate that Mfb1p is a novel, Num1p-independent mitochondrial retention factor that is required for site-specific anchorage of mitochondria at the mother tip. As Mfb1p localizes to mitochondria in the mother tip, it is possible that Mfb1p has a direct role in this process.

Next, we asked whether anchorage of mitochondria in the mother tip depends on interactions with other cellular organelles. The yeast cell cortex contains extensive sheets of cortical endoplasmic reticulum (cER) and plasma membrane as well. Previous studies indicate that Num1p mediates anchorage of mitochondria to the plasma membrane of yeast mother cells^19^,^20^. On the other hand, mitochondria in the bud tip are anchored to cER^14^. To determine whether mitochondria that are anchored to the mother cell tip are anchored to cER at that site, we assessed mitochondrial morphology and distribution in cells that carry deletions in six genes (*IST2*, *TCB1/2/3*, *SCS2*, and *SCS22*) that collectively tether ER to the plasma membrane^27^. We confirmed that deletion of all six genes results in loss of ER from the cortex. However, despite the collapse of cER, accumulation of mitochondria in the mother cell tip and mitochondrial morphology in mother cells are not affected (Supplementary Fig. 3a). Similarly, ER localization is normal in *mfb1Δ* and *num1Δ* cells (Supplementary Fig. 3b). Thus, anchorage of mitochondria to the mother cell tip is independent of cER and probably occurs via interactions between mitochondria and the plasma membrane or other structures at the mother tip.

### Role for Mfb1p mitochondrial quality control and lifespan

**Figure 3:**
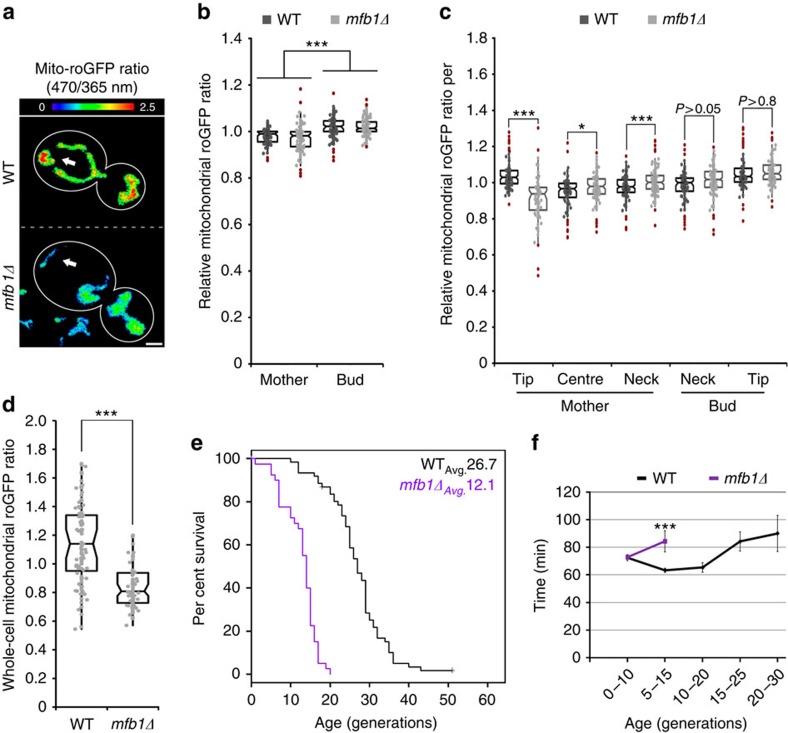
Mfb1p is required for enrichment of high-functioning mitochondria in the mother cell tip and normal mitochondrial function and RLS. (**a**–**d**) Cells expressing mito-roGFP1 were grown to mid-log phase and visualized by fluorescence microscopy as described in Methods. (**a**) Representative images of reduced/oxidized mito-roGFP1 ratios in wild-type (WT) and *mfb1Δ* cells. Higher numbers and warmer colours indicate more reducing mitochondria. Arrows highlight mitochondria at the mother tip. Cell outlines are shown in white; scale bar, 1 μm. (**b**,**c**) Comparison of the reduced/oxidized mito-roGFP1 ratio, normalized to the whole-cell ratio, in WT and *mfb1Δ* cells, between (**b**) mother and bud, and (**c**) five zones of the cell as defined in Fig. 1b (*n*>40 cells per strain). (**d**) Comparison of absolute mito-roGFP1 ratio between WT and *mfb1Δ* cells (*n*>40 per strain). Central bands in the boxes represent the median, boxes indicate the middle quartiles and whiskers extend to the 5th and 95th percentiles; red dots indicate data points beyond this range. All data are representative of three independent experiments. (**e**) Kaplan–Meier survival plot of RLS of WT and *mfb1Δ* mother cells. Cells were grown to mid-log phase, arranged via micro-manipulation and RLS of newborn cells was determined from their first division as described in Methods (*n*>40 for each strain in 2 independent experiments). (**f**) Mean generation time was calculated as the number of minutes between cell divisions during the RLS experiments for WT and *mfb1Δ* cells of different replicative ages. Error bars represent s.e.m. For **e**, statistical significance was determined using the log-rank test: WT versus *mfb1Δ*, *P*<10^−11^. For other panels, statistical significance was determined using Student's *t*-test; *P<0.05, **P<0.01 and ***P<0.005.

As higher-functioning mitochondria are anchored in the mother cell tip and Mfb1p is required for this region-specific anchorage event and also for normal yeast growth, we used mito-roGFP1 to determine whether deletion of *MFB1* affects mitochondrial function in mother cells. Interestingly, deletion of *MFB1* does not inhibit inheritance of fitter mitochondria by yeast daughter cells: mitochondria in the bud are more reduced compared with mitochondria in all regions of the mother cell, in both wild-type and *mfb1Δ* cells (Fig. 3a–c). However, in *mfb1Δ* cells, residual mitochondria in the mother cell tip are no longer higher functioning compared with other mitochondria in the mother cell (Fig. 3a,c) and overall mitochondrial function in the mother cell is significantly lower than that of mitochondria in wild-type cells (Fig. 3d). Thus, although Num1p supports anchorage of a few residual mitochondria at the mother tip, it is insufficient for the anchorage of higher-functioning mitochondria at that site. Instead, Mfb1p is necessary to sustain anchorage of high-functioning mitochondria in the mother tip and this process is required for overall mitochondrial function in yeast mother cells.

As mitochondria are ageing determinants, we asked whether *MFB1* has a role in yeast lifespan. Ageing studies in yeast can model two distinct forms of cellular ageing^28^. Chronological lifespan, the survival time of stationary-phase, non-dividing yeast cells, is a model for stress resistance in postmitotic cells. RLS, the number of times that a cell can divide before senescence, is a model for ageing of division-competent cells. We find that *mfb1Δ* cells exhibit a severely shortened RLS (Fig. 3e). Specifically, the average RLS of wild-type and *mfb1Δ* cells is 26.7 and 12.1 generations, respectively.

Next, we tested the effect of deletion of *MFB1* on yeast healthspan. Early studies revealed that mean generation time of a yeast cell increases with age and is a general indicator of cellular healthspan^29^,^30^. We find that ageing *mfb1Δ* cells have a longer mean generation time compared with age-matched wild-type cells, indicating that Mfb1p is required for normal yeast healthspan (Fig. 3f). Collectively, these results suggest that Mfb1p is critical for yeast lifespan and healthspan, potentially through its function in retention of a high-quality mitochondrial population in mother cells.

### *MMR1* deletion rescues defects in *mfb1Δ* cells

**Figure 4:**
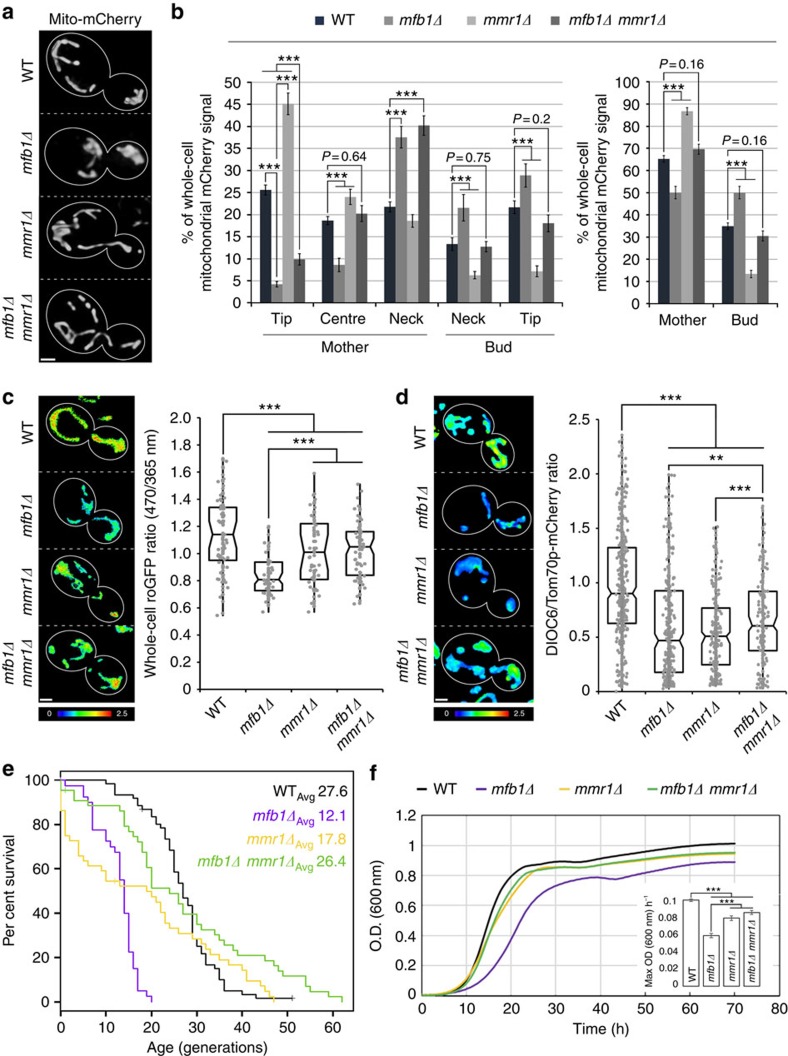
Deletion of *MMR1* rescues defects in mitochondrial inheritance and restores cellular fitness in *mfb1Δ* cells. (**a**,**b**) Cells expressing Cit1p-mCherry were grown to mid-log phase and visualized by fluorescence microscopy. (**a**) Representative 3D renderings of wild-type (WT), *mfb1Δ*, *mmr1Δ* and *mfb1Δ mmr1Δ* cells. (**b**) Relative mitochondrial Cit1p-mCherry intensity distribution in WT, *mfb1Δ*, *mmr1Δ* and *mfb1Δ mmr1Δ* cells in regions as defined in Fig. 1b. The data shown for WT and *mfb1Δ* cells are the same as in Fig. 2d. Data (*n*>40 for each strain) are representative of three independent experiments. (**c**) Mid-log-phase cells expressing mito-roGFP1 were visualized by fluorescence microscopy. Panels show representative images (left) and quantification (right) of the whole-cell reduced/oxidized mito-roGFP1 ratio in WT, *mfb1Δ*, *mmr1Δ* and *mfb1Δ mmr1Δ* cells; higher numbers and warmer colours indicate more reducing mitochondria (*n*>40 for each strain). Data are representative of three independent trials. (**d**) Mid-log-phase cells expressing endogenously tagged Tom70p-mCherry were labelled with DiOC_6_ and relative ΔΨ was determined from the DiOC_6_/Tom70p-mCherry intensity ratio as described inMethods. Panels show representative images (left) and quantification (right) of the whole-cell DiOC_6_/Tom70p-mCherry ratio; (*n*>200 from independent 3 trials); higher numbers and warmer colours indicate higher ΔΨ. (**e**) Kaplan–Meier survival plot showing RLS in WT, *mfb1Δ*, *mmr1Δ* and *mfb1Δ mmr1Δ* cells. Data shown for WT and *mfb1Δ* cells are the same as in Fig. 3e. (**f**) Growth curves for WT, *mmr1Δ*, *mfb1Δ* and *mfb1Δ mmr1Δ* cells in SC medium at 30 °C. Maximum growth rates were calculated for intervals of 2 h. (**b**,**f**) Error bars are s.e.m. (**c**,**d**) Central bands in the boxes represent the median, boxes indicate the middle quartiles and whiskers extend to the 5th and 95th percentiles; red dots indicate points beyond this range. For all images, cell outlines are shown in white; scale bars, 1 μm. Statistical significance was determined using the Wilcoxon rank-sum test (**d**) and the log-rank test for **e** (WT versus *mfb1Δ*, *P*<10^−11^; WT versus *mfb1Δ mmr1Δ*, *P*=0.259; *mfb1Δ* versus *mfb1Δ mmr1Δ*, *P*<10^−9^; *mmr1Δ* versus *mfb1Δ mmr1Δ*, *P*<0.05) or the Student's *t*-test (**b**,**c**,**f**). *P<0.05, **P<0.01 and ***P<0.005.

We hypothesized that the impaired mitochondrial quality and premature ageing of *mfb1Δ* cells might be due to the disproportionate transfer of high-quality mitochondria into daughter cells. If this is the case, then inhibition of mitochondrial inheritance to daughter cells should rescue mitochondrial and cellular fitness in *mfb1Δ* cells. To test this hypothesis, we took advantage of previous observations that deletion of *MMR1* and the associated defect in anchorage of mitochondria in the bud tip and/or transport of mitochondria from mother cells to buds results in increased accumulation of mitochondria in mother cells^10^. We find that deletion of *MMR1* in *mfb1Δ* cells does not restore mitochondrial accumulation specifically at the mother tip (Fig. 4a,b). However, the overall distribution and the partitioning of mitochondria between mother and bud in *mfb1Δ mmr1Δ* cells are similar to those observed in wild-type cells (Fig. 4a,b).

To test whether *MMR1* deletion also restores mitochondrial quality in *mfb1Δ* cells, we assessed mitochondrial redox state via mito-roGFP1. In addition we employed DiOC_6_, a fluorescent dye that responds to mitochondrial membrane potential^31^, as an additional indicator of mitochondrial quality (Supplementary Fig. 4a). To control for variations in total mitochondrial mass, we normalized DiOC_6_ fluorescence to that of Tom70p-mCherry, a protein that is imported into mitochondria in a membrane potential-independent manner^32^. We found that restoring mitochondrial distribution in *mfb1Δ* cells by deleting *MMR1* also improved overall mitochondrial redox state and membrane potential (Fig. 4c,d).

Finally, to determine whether the observed increase in mitochondrial function also affects cellular fitness, we studied the growth rates and RLS of *mmr1Δ mfb1Δ* cells. Deletion of *MMR1* in a wild-type background results in two populations of yeast cells. One population exhibits extended RLS and has mitochondria that are higher functioning compared with those observed in wild-type cells. The other population ages prematurely and has lower-functioning mitochondria^10^. We find that there is no short-lived population of *mfb1Δ mmr1Δ* cells. Moreover, we find that double-mutant cells exhibit an RLS that is longer than that of *mfb1Δ* cells (Fig. 4e). Thus, deletion of *MFB1* extends RLS in *mmr1Δ* cells and vice versa. Deletion of *MMR1* in *mfb1Δ* cells also rescued cellular growth rates (Fig. 4f). Overall, these results indicate that Mfb1p and Mmr1p have antagonistic effects in controlling mitochondrial partitioning, and that the disproportionate loss of mitochondria from mother cells is the primary cause for cellular growth defects and premature ageing in *mfb1Δ* cells.

### Association of Mfb1p with mitochondria to the bud tip

We next asked how the Mfb1p-dependent mitochondrial anchorage site is established. It is possible that Mfb1p binds preferentially to higher-functioning mitochondria, so that localization of the protein to the mother cell tip results in an enrichment of high-quality mitochondria at that site. If so, then treatments that compromise mitochondrial quality should abolish Mfb1p-dependent mitochondrial anchorage at the mother tip. Indeed, previous results suggest that endogenous pathways of mitochondrial quality control are sensitive to the loss of membrane potential and to mitochondrial oxidative stress^33^,^34^. Therefore, we treated cells with paraquat (PQ), an agent that generates superoxide in mitochondria and other cellular compartments^35^,^36^, at concentrations that render mitochondria more oxidized and inhibit, but do not abolish, growth (data not shown). We also treated cells with the proton ionophore carbonyl cyanide-4-(trifluoromethoxy)phenylhydrazone (FCCP), under conditions that results in loss of membrane potential (Supplementary Fig. 4a). Neither acute nor chronic exposure to PQ nor treatment with FCCP affected Mfb1p-dependent mitochondrial anchorage (Supplementary Fig. 5a,c) or localization of Mfb1p to the mother cell tip (Supplementary Fig. 5a,d–g). Therefore, binding of Mfb1p to mitochondria is not affected by mitochondrial membrane potential or ROS levels.

Instead, we find that Mfb1p undergoes cell cycle-regulated localization to sites of anchorage and accumulation of higher-functioning mitochondria. Our previous studies revealed that higher-functioning mitochondria that are more reducing and contain less ROS are anchored in the yeast bud tip^10^. Using DiOC_6_ and Tom70p-mCherry, we find that mitochondria in the bud tip also have a higher membrane potential compared with mitochondria in the mother cell (Fig. 4d).

**Figure 5:**
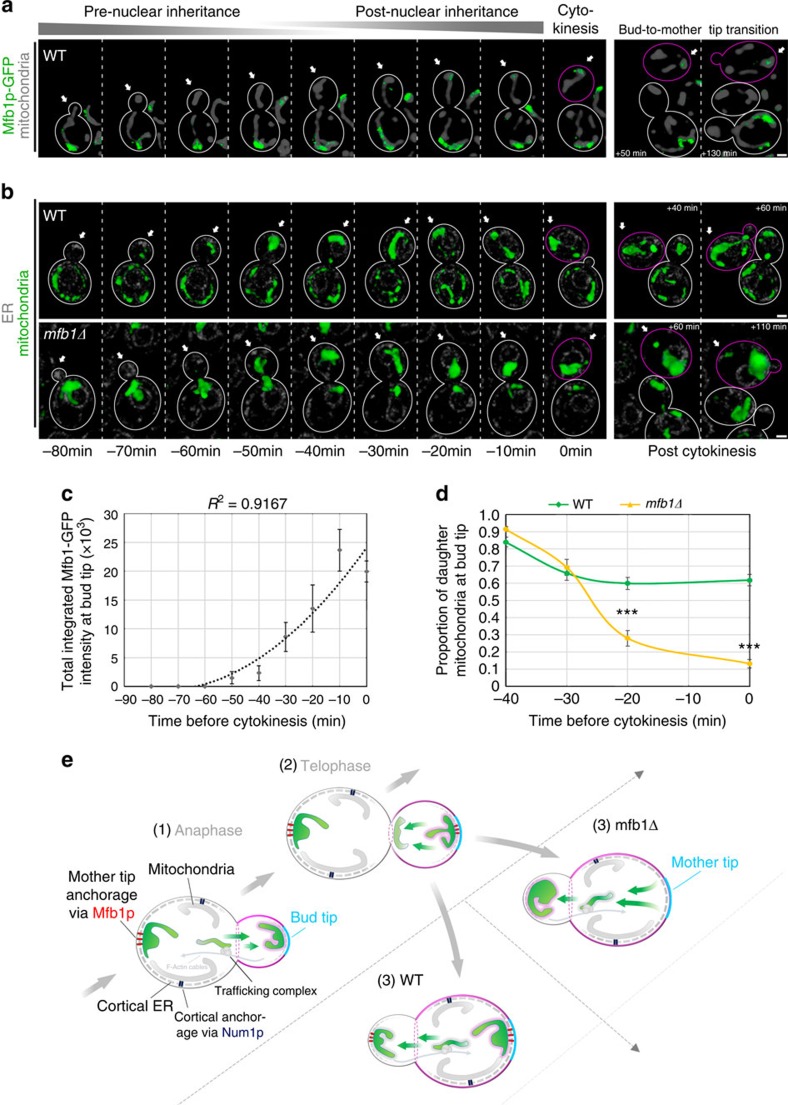
The Mfb1p-dependent mitochondrial retention site is established within the developing bud in late M-phase. (**a**,**b**) Representative time-lapse series of mitochondrial inheritance. Cells expressing Cit1p-mCherry and either (**a**) Mfb1p-GFP or (**b**) Pho88p-GFP were grown to mid-log phase, loaded into a CellASIC microfluidic chamber and imaged at 10-min intervals for 12 h under perfusion with SC medium. White arrows indicate the bud tip that turns into the mother tip during BTMT in the newborn cell (purple outline). Maternal cell outlines are shown in white. Scale bars, 1 μm. (**a**) The time course of mitochondrial and Mfb1p-GFP distribution was visualized by fluorescence microscopy in wild-type (WT) cells. (**b**) In WT and *mfb1Δ* cells, mitochondria and the ER marker Pho88p-GFP were visualized by fluorescence microscopy. Pho88p-GFP allows observation of nuclear inheritance at *ca*. −40 min. (**c**) The accumulation of Mfb1p-GFP at the tip of the developing bud was quantified as total intensity (*n*>30 in 3 trials). Data were fitted to a quadratic polynomial (dotted line). (**d**) Quantification of the relative distribution of mitochondrial Cit1p-mCherry signal as a function of time between bud neck and bud tip as defined in Fig. 1b in WT and *mfb1Δ* cells (*n*>40). Data are representative of three independent experiments. (**e**) A model of Mfb1p-mediated mitochondrial retention: (1) high-functioning mitochondria (green) are partitioned between mother and bud tip, by Mfb1p-dependent anchorage at the mother and anterograde trafficking of mitochondria along actin cables into the emerging bud (purple outline). (2) Post-nuclear inheritance: Mfb1p accumulates and partially retains mitochondria at the bud tip. (3) WT: Mfb1p maintains a high-functioning mitochondrial population throughout BTMT and following division cycles. *mfb1Δ*: in absence of Mfb1p, mother-tip-specific mitochondrial retention fails and high-functioning mitochondria are disproportionally lost to daughter cells. Error bars represent s.e.m. Statistical significance was determined using Student's *t*-test; *P<0.05, **P<0.01 and ***P<0.005. Cell outlines are shown in white.

Surprisingly, our long-term time-lapse imaging revealed that some Mfb1p localizes to the bud tip at late stages in the cell division cycle. Although the majority of Mbf1p localizes to the mother cell tip, we detect some Mfb1p in the bud tip after migration of the nucleus from the mother cell to the bud, roughly 40 min before cytokinesis. We also observe an increase in the amount of Mfb1p in the bud tip with time as cells progress towards cytokinesis (Fig. 5a,c). As higher-functioning mitochondria are anchored in the bud tip, localization of Mfb1p to that site late in the cell cycle may allow Mfb1p to interact specifically with higher functioning mitochondria.

Interestingly, we find that this localization of Mfb1p to the bud tip late in the cell cycle also contributes to its localization in the mother cell tip during the next yeast cell generation. In haploid yeast cells, new buds emerge adjacent to the site of the previous cytokinesis. One consequence of this axial budding pattern is that the bud tip of a haploid daughter cell becomes the mother cell tip when that daughter cell becomes a mother cell^37^,^38^. We will refer to this well-characterized event as bud-to-mother-tip transition (BTMT). BTMT is readily detected by monitoring Mfb1p localization in long-term time-lapse imaging. Mfb1p that localizes to the bud tip late in the cell division cycle persists at that site as the bud tip becomes the mother tip after the bud separates from its mother cell and undergoes the next round of cell division (Fig. 5b,e). Thus, localization of Mfb1p to the bud tip late in the cell division cycle places it at the site that will become the mother tip after the BTMT.

Finally, we asked whether Mfb1p at the bud tip also functions in anchorage of mitochondria at that site late in the cell cycle. Our long-term time-lapse imaging revealed that 40% of the mitochondria that are anchored in the bud tip are released from that site at 30 min before cytokinesis (Fig. 5b,d). As release of mitochondria from the bud tip and localization of Mfb1p to the bud tip occur at roughly the same time in the cell cycle, we tested whether Mfb1p is required for retention of some mitochondria in the bud tip. Indeed, we find that deletion of *MFB1* causes additional release of mitochondria from the yeast bud tip. In contrast to wild-type cells, where only 40% of the mitochondria are released, 86% of the mitochondria are released from the bud tip in *mfb1Δ* cells (Fig. 5b,d). Together, these findings indicate that an Mfb1p-dependent mitochondrial retention site is established at the bud tip before cytokinesis and are consistent with the notion that mother tip-specific enrichment of high-quality mitochondria originates from Mfb1p-dependent capture of higher-functioning mitochondria in the bud tip during late stages in the cell division cycle.

## Discussion

Previous studies indicate that mitochondria are anchored in the yeast bud tip, and that this process is required for mitochondrial inheritance, mitochondrial quality control during inheritance and yeast lifespan control^7^,^11^. Other studies indicate that mitochondria also accumulate in the tip of the mother cell distal to the bud^15^. Here we observe that mitochondria that accumulate in the mother cell tip are under tension and are therefore anchored at that site. Finally, although mitochondria are anchored in the bud tip by association with cER, which itself is anchored to the plasma membrane^14^, we find that cER is not required for anchorage of mitochondria in the maternal cortex or the mother cell tip. Thus, mitochondrial anchorage in the mother cell presumably occurs through the plasma membrane or some other structures at the mother tip.

Intriguingly, we found that mother-tip-specific anchorage is not affected by deletion of Num1p, a protein that mediates cortical anchorage of mitochondria throughout the mother cell^19^,^20^. Instead, we here identify a novel role for Mfb1p in sustaining site-specific accumulation and anchorage of mitochondria at the mother tip of budding yeast cells. We find that Mfb1p localizes to mitochondria that are anchored in the mother cell tip. Moreover, deletion of *MFB1* results in loss of 87% of the mitochondria from the mother cell tip. Conversely, Num1p-mediated anchorage to the mother cell cortex occurs normally in *mfb1Δ* cells. We thus confirm the role of Num1p in cortical mitochondrial anchorage and find that Mfb1p is required for site-specific mitochondrial anchorage at the mother tip. As of yet, the mechanisms for Mfb1p-dependent mother-tip-specific mitochondrial anchorage remain to be identified. As Mfb1p localizes to mitochondria in the mother cell tip, it is possible that Mfb1p plays a direct role in anchorage of the organelle at this site. Together, Num1p and Mfb1p largely explain mitochondrial anchorage and retention in the mother cell in *S. cerevisiae*.

The evolution of two independent maternal anchorage mechanisms in yeast strongly suggests distinct functions for these two systems. Intriguingly, we found that the mother cell tip mitochondria are less oxidized than those elsewhere in the mother cell. This finding was surprising, as mitochondria, both in yeast and in mammalian cells, undergo fusion and fission cycles, which suggests that any qualitative differences should dissipate through content mixing^39^. However, as mitochondria in mother cells are functionally distinct, they must also be physically distinct. Indeed, previous studies indicate that messenger RNA for *DNM1*, the mitochondrial fission gene, is enriched in the bud tip and relies on the mRNA transport adapter She2p for that localization^40^. Therefore, it is possible that mitochondrial fusion and/or fission may occur in a region-specific manner.

Strikingly, we find that a population of mitochondria that is highly reduced and therefore higher functioning is anchored in the mother cell tip. We also find that deletion of *MFB1* results in defects not just in anchorage of mitochondria in the mother cell tip, but in the enrichment of higher-functioning mitochondria at that site. Specifically, we find that residual mitochondria that undergo Num1p-dependent anchorage at the mother tip in *mfb1Δ* cells are not high-functioning. Thus, our studies support a role for Num1p in cortical anchorage of mitochondria, but not in mitochondrial quality control. In contrast, we find that Mfb1p is necessary to sustain not only quantity but also quality of maternal mitochondria and does so in a region-specific manner.

As mitochondria are established ageing determinants in yeast and other eukaryotes, we asked whether *MFB1* influences overall mitochondrial fitness, RLS and healthspan. Interestingly, deletion of *MFB1* does not inhibit asymmetric inheritance of mitochondria: fitter mitochondria are still anchored in the bud tip of *mfb1Δ* cells and are therefore destined for inheritance by *mfb1Δ* daughter cells. However, the deletion of *MFB1* results in an overall decrease in mitochondrial function ans reduced RLS and healthspan. Specifically, mitochondria in *mfb1Δ* cells are more oxidizing and harbour lower membrane potential compared with mitochondria in wild-type cells. Moreover, deletion of *MFB1* results in a 50% decrease in cellular RLS and rapid increase in mean generation time at all but the first stages of their RLS.

To determine whether the defects in mitochondrial function and cellular lifespan and healthspan are due to the disproportionate loss of mitochondria to daughter cells, we tested whether inhibiting mitochondrial inheritance in *mfb1Δ* cells could rescue mitochondrial function and cellular lifespan. Previous studies indicate that deletion of *MMR1* inhibits mitochondrial inheritance by inhibiting anchorage of mitochondria in the bud tip and/or transport of mitochondria across the bud neck^14^,^24^,^25^,^26^. We find that deletion of *MMR1* rescues mitochondrial distribution, mitochondrial quality and cellular ageing defects in *mfb1Δ* cells. Furthermore, we find that deletion of *MFB1* prevents premature ageing observed in *mmr1Δ* single mutants. Together, these results suggest that defects in cellular RLS and mitochondrial fitness in *mfb1Δ* cells are primarily due to mitochondrial inheritance defects. Specifically, our findings suggest that Mfb1p is critical for balanced partitioning of high-functioning mitochondria: ensuring that buds receive fitter mitochondria, while avoiding depletion of all high-functioning mitochondria from mother cells.

We find no evidence for selective binding of Mfb1p to higher-functioning mitochondria. However, as described above, higher-functioning mitochondria are preferentially inherited by yeast daughter cells and are retained in the daughter cell by anchorage to the yeast bud tip. We find that some Mfb1p localizes to the bud tip and is required for bud-tip anchorage of mitochondria during late stages in the cell cycle.

We also obtained evidence that the cell polarity machinery of yeast contributes to Mfb1p localization and function in anchorage of higher-functioning mitochondria in mother cells. In haploid yeast, newborn cells bud at sites adjacent to the site of the previous cytokinesis^37^,^38^. As a result, the bud tip in the developing daughter cell becomes the mother tip in the mature cell. Thus, localization of Mfb1p to the bud tip at the end of the cell division cycle places it and its associated higher-functioning mitochondria at the presumptive mother cell tip in the next round of cell division. Based on these results, we propose that the specific enrichment of highly reduced mitochondria at the tip of mature mother cells originates in their early capture, first by Mmr1p and later by Mfb1p, during development of the bud. Conversely, we suggest that deletion of *MFB1* overrides the rejuvenating effects of asymmetric mitochondrial inheritance, as high-quality mitochondria cannot be retained in future cell divisions and premature ageing occurs (Fig. 5e).

An open question that remains is whether and how Mfb1p in wild-type cells may contribute to mitochondrial quality control and cellular lifespan, beyond the initial retention of high-quality mitochondria in mother cells during the first cell divisions. As the fundamental polarity of haploid yeast cells (unipolar budding) remains generally intact with age^38^, it is possible that the mother cell tip and Mfb1p-dependent mitochondrial anchorage at this site persist throughout the cell's RLS. As a result, Mfb1p-dependent anchorage may continue to ensure the partial retention of high-quality mitochondria during cell division as cells age. Interestingly, mother cells must replenish their mitochondrial population after each cell division. As mitochondria are only produced from pre-existing mitochondria, mitochondria derived from high-functioning mitochondria that were retained by Mfb1p will also be higher functioning. Thus, there are multiple mechanisms whereby Mfb1p can affect lifespan in *S. cerevisiae*.

Overall, our results support a role for the non-canonical F-box protein Mfb1p in region-specific retention of a high-functioning pool of mitochondria in the tip of mother cell distal to the bud. They also indicate that this process is required for mitochondrial quality control during inheritance and for normal RLS and healthspan in budding yeast cells. As Mfb1p localizes to mitochondria that are anchored in the mother cell tip and has the capacity to bind to mitochondria, it is possible that it plays a direct role in this anchorage process. Finally, although Mfb1p localizes to mitochondria that are anchored in the mother cell tip throughout the cell cycle, we obtained evidence that some Mfb1p localizes to the bud tip late in the cell division cycle. As the bud tip of haploid yeast daughter cells becomes the mother cell tip when those daughters become mother cells, this process may allow the protein to associate with high-functioning mitochondria and anchor those mitochondria in the mother cell tip after the bud to mother transition. Finally, as asymmetric inheritance of mitochondria occurs in mammalian cells and affects cell fate^11^, it is possible that similar mechanisms for region-specific retention of higher-functioning mitochondria during asymmetric cell division occur in other eukaryotes.

## Methods

### Yeast growth conditions

All *S. cerevisiae* strains used in this study were derived from wild-type BY4741 (MAT**a** *his3Δ0*, *leu2Δ0*, *met15Δ0* and *ura3Δ0*) from Open Biosystems (Huntsville, AL), unless otherwise specified. Yeast cells were propagated and manipulated as previously described^41^. With the exception of experiments for the determination of RLS, all cells were grown at 30 °C with shaking in synthetic complete (SC) medium, unless otherwise stated. For strains requiring selection, appropriate SC dropout medium was used. For imaging experiments, all strains were grown overnight in liquid culture and diluted 4 h before image acquisition so that cultures were growing in logarithmic phase at the time of imaging.

### Yeast strain construction

Knockout strains (Supplementary Table 1) were created by replacing the target genes with knockout cassettes containing the selectable markers *LEU2*, *URA3* or KanMX6, through homologous recombination according to previously described protocols^42^,^43^. Briefly, PCR fragments containing an appropriate selectable marker flanked by 40 bp of homology to regions immediately upstream and downstream of the target gene open reading frame were amplified using primers listed in Table 2 and plasmids pFA6a-kanMX6, pOM12 or pOM13 (Addgene). Transformations were carried out using the lithium acetate method^42^. Transformants were selected on SC dropout plates for auxotrophic markers or rich yeast extract peptone dextrose (YPD) medium plates containing 200 μg ml^−1^ Geneticin (Sigma-Aldrich, St Louis, MO) to select KanMX6-positive cells. Positive transformants were confirmed by PCR.

To create strains expressing fluorescently tagged fusion proteins such as Cit1-mCherry, we followed the method outlined above; however, PCR fragments were amplified from plasmids pFA6a-GFP(S65T)-kanMX, pCY3090-02, pCY3080-07 (Addgene), pWP63 or pWP64 (available on request) to contain the coding regions for GFP(S65T), mCherry, Citrine, YFP and CFP, followed by kanMX6, hphMX4, zeocin or LEU2, respectively. The homology of the flanking regions was designed to insert the cassette in frame with the target open reading frame's 3′-end. Selection was carried out on YPD plates using 200 μg ml^−1^ Geneticin (for KanMX6) (Sigma-Aldrich) or 300 μg ml^−1^ Hygromycin B (for hphMX4) (Life Technologies, Carlsbad, CA).

### Microscopy

Fluorescence microscopy was carried out on one of the following systems: (1) an inverted AxioObserver.Z1 microscope with a × 100/1.3 oil EC Plan-Neofluar objective (Zeiss, Thornwood, NY), an Orca ER cooled CCD (charge-coupled device) camera (Hamamatsu, Bridgewater, NJ), a metal-halide lamp and an light-emitting diode (LED) Colibri system (Zeiss) including LED wavelengths at 365 and 470 nm; (2) a Nikon A1R MP laser scanning confocal attachment on a Nikon Ti Eclipse inverted microscope using a × 100/1.45 Plan Apo Lambda oil-immersion objective and standard lasers and filters (Nikon Instruments, Melville, NY); (3) a spinning-disk confocal microscope combining a CSU-X1 spinning disk attachment (Yokogawa Electronic Corporation, Tokyo, Japan) on a Nikon Ti Eclipse inverted microscope (Nikon) equipped with an electron multiplying CCD camera (Evolve, Photometrics, Tucson, AZ), 50 mW lasers at 488 and 561 nm, standard emission filters and a CFI Plan Apo × 100 1.45 numerical aperture oil objective (Nikon). System (1) was controlled by ZEN software (Zeiss). Systems (2) and (3) were controlled by NIS Elements Advanced Research software (Nikon). Details are given within the sections below describing each experiment.

### Analysis of mitochondrial distribution

Analysis of mitochondrial distribution was performed as previously described^14^. Cells expressing tagged Cit1p-mCherry from the endogenous locus were imaged on System (1) using a standard rhodamine filter set (Zeiss filter set 43 HE; excitation FT 570, emission 605/70). Images were collected through the entire cell depth (21 *z*-sections at 0.3-μm intervals), using 1 × 1 binning, 200 ms exposure and analogue gain at 216. Images were deconvolved using an iterative restoration algorithm (Volocity, Perkin-Elmer, Waltham, MA). The relative distribution of mitochondrial mass was estimated by determining total mitochondrial voxel intensity in thresholded, deconvolved wide-field *z*-series of budding cells, for three equal regions in mothers and two equal regions in daughter cells, as defined in Fig. 1b. Mother and bud compartments were identified in corresponding transmitted-light images. Cells with buds of diameter smaller than 0.2 μm (measured along the mother-bud axis) were excluded.

### Analysis of mitochondrial motility

To determine frequency and velocity of mitochondrial movements, cells expressing Cit1p-mCherry from the endogenous locus were imaged on System (2). Three-dimensional time series were collected consisting of a *z*-series (3 μm at 0.5-μm intervals) every 1 s for a total of 30 s. Images were deconvolved using an iterative restoration algorithm and analysed in Volocity (Perkin-Elmer). Mitochondrial movement was scored using mitochondrial tips or leading edges of mitochondrial tubules that moved for three consecutive frames in the same direction. Mitochondrial springback events were defined as motility events in which mitochondria that remain associated with the mother cell tip undergo movement in the anterograde direction followed by a retrograde movement of the organelle back to its original position within the 30 s time of imaging.

### Analysis of mitochondrial redox state and mitochondrial membrane potential

For Mito-roGFP1 imaging, select strains were transformed with a centromeric plasmid for constitutive expression of roGFP1 fused to the mitochondrial targeting sequence of ATP9 (refs 10, 18). To assess the functionality and dynamic range of the mito-roGFP1 signal in response to oxidizing and reducing conditions, cells were treated with 5 mM H_2_O_2_ or 5 mM dithiothreitol for 30 min, while shaking at 30 °C, before imaging. For all experiments, mito-roGFP1 imaging was performed on System (1) using alternating excitation by LEDs at 365 and 470 nm, using 150-ms exposure and 25% LED power, and 200-ms exposure and 100% LED power respectively, through a modified GFP filter (Zeiss filter Set 46 HE with excitation filter removed; dichroic FT 515, emission 535/30 nm). Images were collected through the entire cell depth (15 *z*-sections at 0.4-μm intervals) using 1 × 1 binning and analogue gain at 216. Images were deconvolved using an iterative restoration algorithm and analysed in Volocity. The reduced-to-oxidized mito-roGFP ratio was calculated by dividing voxel-by-voxel intensity of the reduced (*λ*ex=470 nm, *λ*em=525 nm) over oxidized (*λ*ex=365 nm, *λ*em=525 nm) channels in Volocity software using background selection and thresholding steps. The resulting ratio channel was measured while excluding zero values. Mother and bud compartments were identified in corresponding transmitted-light images. To assess the relative mitochondrial redox state within different areas of the cell, mothers and buds were divided into five regions of interest according to Fig. 1b. Cells with buds of diameter smaller than 0.2 μm (measured along the mother-bud axis) were excluded.

### Analysis of mitochondrial membrane potential using DiOC_6_

To assess mitochondrial membrane potential, cells expressing Tom70p-mCherry from the endogenous locus were stained with DiOC_6_ (Life Technologies)^4^. Mid-log phase cells were washed once in 10 mM HEPES pH 7.6, containing 5% glucose. Cells were incubated in the same buffer with 17.5 nM DiOC_6_ and 0.1% ethanol for 15 min. Cells were washed three times in buffer and imaged immediately, using System (1). DiOC_6_ fluorescence and Tom70p-mCherry fluorescence were excited by a metal-halide lamp through a GFP filter (Zeiss filter set 46 HE) and a rhodamine filter (Zeiss filter set 43 HE; excitation FT 570, emission 605/70), respectively, using 1 × 1 binning and analogue gain at 216. The entire cell depth was imaged in 15 *z*-slices at 0.5-μm intervals. DiOC_6_ signal dissipates noticeably after as little as 1 min after cells are mounted onto coverslips (Adam L. Hughes, personal communication and data not shown). Therefore, only a single image was collected for each prepared slide. For FCCP treatment, 10 μM FCCP (Sigma-Aldrich) was added during the 15-min DiOC_6_ incubation. Images were deconvolved using an iterative restoration algorithm and analysed in Volocity (Perkin-Elmer). Membrane potential was estimated as the mitochondrial DiOC_6_ fluorescence intensity normalized to mitochondrial mass, by dividing voxel-by-voxel intensity of the DiOC_6_ channel by the Tom70p-mCherry channel. The resulting ratio channel was measured, while excluding zero values. Mother and bud compartments were identified in corresponding transmitted-light images. Cells with buds of diameter smaller than 0.2 μm (measured along the mother-bud axis) were excluded.

### Analysis of Mfb1p sensitivity to mitochondrial stress

Relative mitochondrial and Mfb1p distribution was quantified within regions of interest in cells expressing Tom70p-mCherry and Mfb1p-Citrine from the endogenous loci. The Citrine tag was used, because stress-induced mitochondrial autofluorescence produces high background in cyan fluorescent protein and GFP channels. To disrupt membrane potential, cells were treated for 15 min with 10 μM FCCP. To induce oxidative stress via superoxide^44^, cells were treated with 20 mM PQ (Sigma-Aldrich) for 30 min for acute high-level exposure or with 2.5 mM PQ for 12 h for chronic low-level exposure in shaking SC liquid culture at 30 °C.

### RLS analysis

RLS measurements were performed as described previously^45^, without α-factor synchronization. Briefly, frozen glycerol stocks of select strains (stored at −80 °C) were streaked out on YPD plates and grown for 2 days. Single colonies were grown overnight in liquid YPD at 30 °C, diluted and grown to exponential phase for 4 h in YPD at 30 °C. Two microlitres of the cell suspension was streaked onto a YPD plate and small-budded cells were isolated and arranged in a matrix using a micromanipulator mounted onto a dissecting microscope (Zeiss). On completion of budding, mother cells were discarded; the time and number of divisions of the corresponding daughter cells was recorded until all replication ceased.

### Long-term fluorescence microscopy

Long-term imaging of yeast cells over multiple rounds of cell division was performed by loading cells growing in mid-log phase into a CellASIC (EMD Millipore, Billerica, MA) microfluidic flow chamber (Y04C plate) controlled by the ONIX Control System and software (EMD Millipore). The chamber was kept at 30 °C and cells were perfused with SC medium. The chamber was mounted on System (3). Strains expressing Cit1-mCherry and either Pho88-GFP or Mfb1-GFP, respectively, were imaged using excitation at 561 nm (mCherry) and 488 nm (GFP). Three-dimensional time series were collected consisting of a *z*-series (3 μm at 0.5-μm intervals) every 10 min for a total of 12 h. Images were deconvolved using an iterative restoration algorithm and analysed in Volocity (Perkin-Elmer).

### Statistical methods

Statistical tests were performed in Microsoft Excel or R-3.1.0. According to data structure, *P*-values were determined using appropriate variants of the Student's *t*-test for normal distributions, Wilcoxon rank-sum test for non-parametric data, log-rank test for RLS data and Fisher's exact test for count data (see figure legends for details and significance thresholds). Sample sizes were chosen based on a qualitative assessment of variability and reproducibility for each individual experiment.

## Additional information

**How to cite this article:** Pernice, W. M. *et al*. A role for Mfb1p in region-specific anchorage of high-functioning mitochondria and lifespan in *Saccharomyces cerevisiae*. *Nat. Commun.* 7:10595 doi: 10.1038/ncomms10595 (2016).

## Supplementary Material

Supplementary Information Supplementary Figures 1-5 and Supplementary Tables 1-2
