# Supplementary material for: A role for Mfb1p in region-specific anchorage of high-functioning mitochondria and lifespan in Saccharomyces cerevisiae
Source: Nat Commun. 2016 Feb 3;7:10595. doi: 10.1038/ncomms10595 (PMC4742906; doi:10.1038/ncomms10595)
Supplement: Supplementary Information — Supplementary Figures 1-5 and Supplementary Tables 1-2 [file ncomms10595-s1.pdf]

Supplementary Figures:

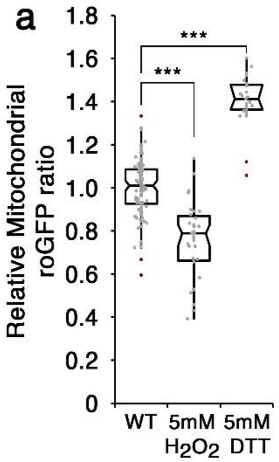

Supplementary Figure 1: roGFP allows ratiometric assessment of mitochondrial redox state a) Notched box plot of the average reduced/oxidized mito-roGFP1 ratio in WT cells treated with H_2O_2 (5 mM) or DTT (5 mM) relative to untreated cells. n > 40 cells per condition. The central band in the box represents the median, boxes indicate the middle quartiles, and whiskers extend to the 5th and 95th percentiles; red dots indicate data points beyond this range. Statistical significance was determined using the Student's *t*-test. * $p < 0.05$, ** $p < 0.01$, *** $p < 0.005$.

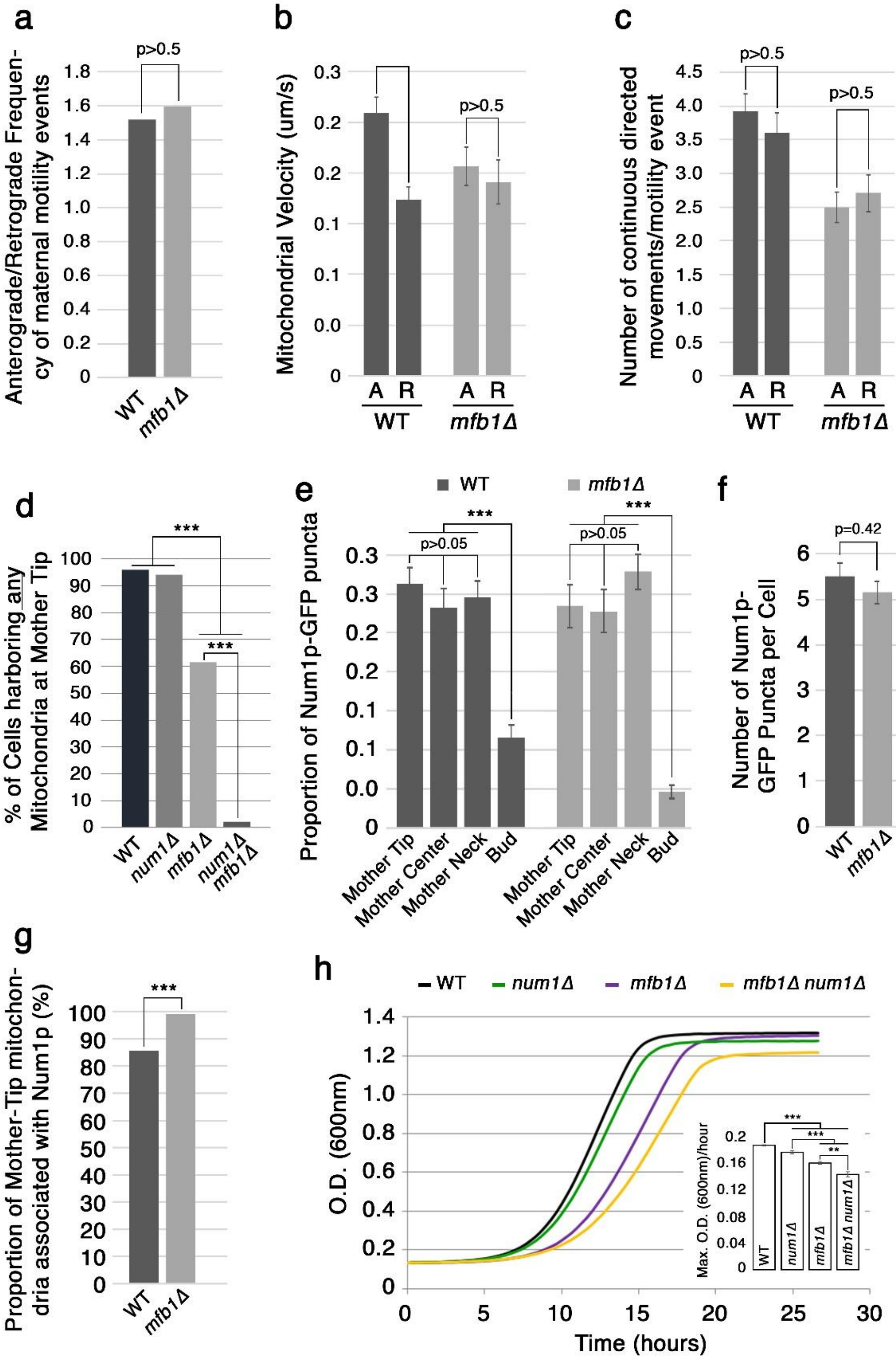

Supplementary Figure 2: Independent function of Num1p and Mfb1p a-c. To test for changes in motility upon deletion of MFB1 that could explain the lack of mitochondrial accumulation at the mother tip (either increased anterograde trafficking or decreased retrograde trafficking), **a)** ratio of frequency of anterograde events to that of retrograde events, **b)** velocity and **c)** processivity of anterograde and

retrograde mitochondrial motility events were measured in cells expressing Cit1p-mCherry. Z-series were collected every 1 s for 30 s and analyzed as described in Experimental Procedures. **d)** The prevalence of mitochondrial localization to the mother tip was quantified in WT, *num1Δ*, *mfb1Δ* and *num1Δ mfb1Δ* cells expressing Cit1p-mCherry (n > 80). **e)** Relative Num1p-puncta distribution in WT and *mfb1Δ* cells expressing Num1p-GFP in 3 areas of the dividing mother cell (see Fig.1b) and the bud. **f)** Total number of Num1p-GFP puncta per cell. **g)** Proportion of WT and *mfb1Δ* cells displaying Cit1p-mCherry at the mother tip in which in which colocalized Num1p-GFP puncta were also observed (n > 80). **d-g.** Data is representative of three independent experiments. **h)** Growth of WT, *num1Δ*, *mfb1Δ* and *num1Δ mfb1Δ* cells in YPD medium at 30°C. Maximum growth rates were calculated for intervals of 2h. Error bars represent standard errors of the mean. Statistical significance was determined using Fisher's exact test (d,g) or Student's *t*-test. * $p < 0.05$, ** $p < 0.01$, *** $p < 0.005$.

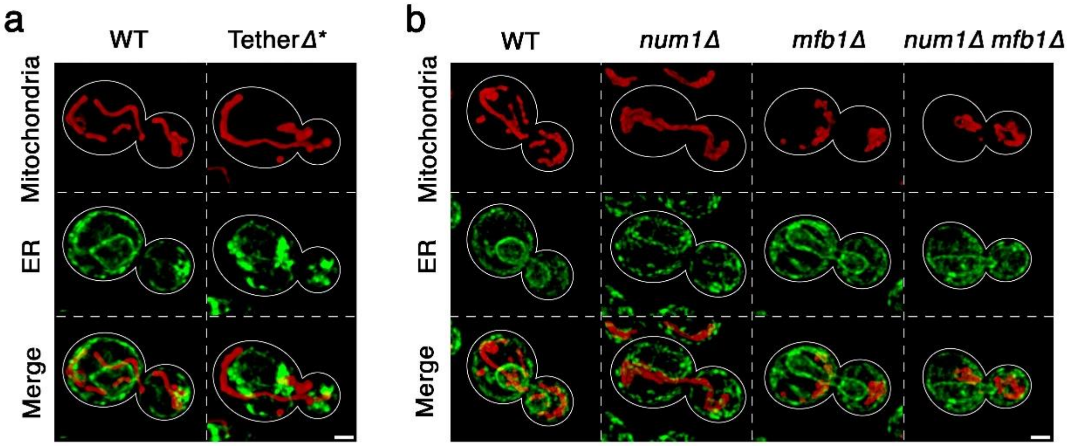

Supplementary Figure 3: Mitochondrial tethering occurs independent of cER a) Mitochondria accumulate at the mother tip despite loss of of cortical ER. Representative 3D renderings of WT and TetherΔ (*Ist2Δ, Tcb1/2/3Δ, Scs2Δ and Scs22Δ*) cells expressing Cit1p-mCherry (mitochondria) and Pho88-CFP and Pho88-YFP (ER) respectively. **b)** Cortical ER is present at mother cell retention sites in m*fb1Δ* and *num1Δ* cells even when mitochondria fail to accumulate there. Representative 3D renderings of WT, *num1Δ*, *mfb1Δ* and *num1Δ mfb1Δ* cells expressing Cit1p-mCherry (mitochondria) and Pho88p-GFP (ER). **a-b.** Cell outlines are shown in white; scale bars = 1 µm.

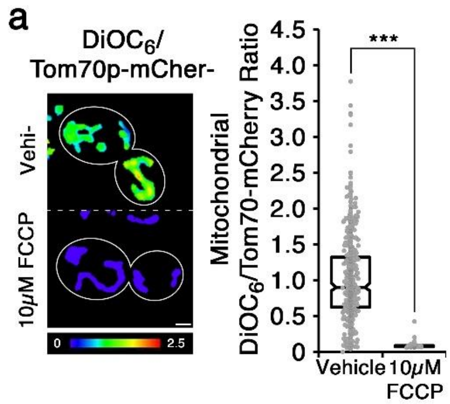

Supplementary Figure 4: The DiOC$_6$/Tom70p-mCherry ratio allows assessment of mitochondrial membrane potential a) Mid-log-phase cells expressing Tom70p-mCherry were labeled with DiOC$_6$ as described in Experimental Procedures. To determine the sensitivity of the DiOC$_6$/Tom70p-mCherry ratio to mitochondrial membrane potential, cells were treated with 10 µM FCCP or vehicle (DMSO). Panels show representative images and quantitation of the whole-cell mitochondrial DiOC$_6$/Tom70p-mCherry ratio ($n > 40$); warmer colors and higher numbers indicate higher mitochondrial membrane potential. The central band in the box represents the median, boxes indicate the middle quartiles, and whiskers extend to the 5th and 95th percentiles; red dots indicate data points beyond this range. Statistical significance was determined using the Wilcoxon Rank-Sum test. * $p < 0.05$, ** $p < 0.01$, *** $p < 0.005$. Cell Outlines are shown in white.

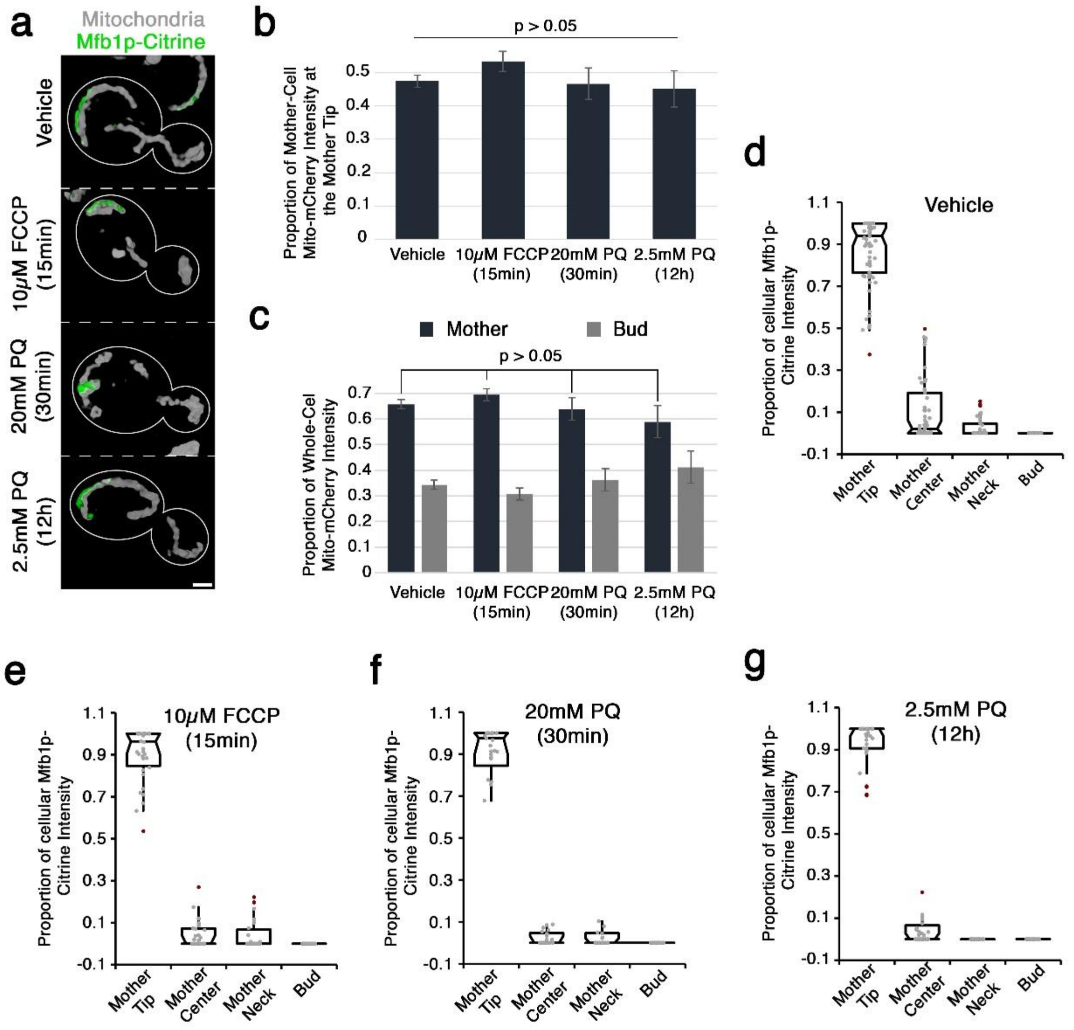

Supplementary Figure 5: Mfb1p localization is not sensitive to oxidative stress or loss of mitochondrial membrane potential a-g . Cells expressing Tom70p-mCherry and Mfb1-Citrine were grown to mid-log phase, treated with vehicle or drug, and visualized by fluorescence microscopy. **a)** Representative 3D renderings of mitochondria and Mfb1p-Citrine after treatment with FCCP or acute or chronic paraquat (PQ). Cell outlines are shown in white. Scale bars = 1 µm. **b)** Cit1p-mCherry intensity at the mother tip, expressed as a proportion of the total intensity in the mother cell. **c)** Cit1p-mCherry intensity in mother and daughter, expressed as a proportion of the total intensity in the entire cell (mother + daughter), after FCCP and PQ treatment. Error bars indicate standard errors of the mean (n > 40 for each condition). **d-g.** Relative distribution of Mfb1p-Citrine after FCCP and PQ treatment (n > 40 for each condition). The central band in the box represents the median, boxes indicate the middle quartiles, and whiskers extend to the 5th and 95th percentiles; red dots indicate data points beyond this range.Statistical significance was determined using Student's *t*-test. * $p < 0.05$, ** $p < 0.01$, *** $p < 0.005$.

Supplementary Tables:

Supplementary Table 1: Strains used in this study

Strains	Genotype	Source
BY4741	*MAT**a** his3Δ1 leu2Δ0 met15Δ0 ura3Δ0*	Open Biosystems
WPY0005	*MAT**a** his3Δ1 leu2Δ0 met15Δ0 ura3Δ1 CIT1-mCherry-hphMX4*	This Study
WPY0006	*MAT**a** his3Δ1 leu2Δ0 met15Δ0 ura3Δ2 CIT1-mCherry-hphMX4 mfb1Δ::kanMX6*	This Study
WPY0007	*MAT**a** his3Δ1 leu2Δ0 met15Δ0 ura3Δ3 CIT1-mCherry-hphMX4 mmr1Δ::LEU2*	This Study
WPY0008	*MAT**a** his3Δ1 leu2Δ0 met15Δ0 ura3Δ5 CIT1-mCherry-hphMX4 mfb1Δ::kanMX6 mmr1Δ::LEU2*	This Study
WPY0013	*MAT**a** his3Δ1 leu2Δ0 met15Δ0 ura3Δ5 CIT1-mCherry-hphMX4 PHO88-GFP(S65T)-KanMX6*	This Study
WPY0014	*MAT**a** his3Δ1 leu2Δ0 met15Δ0 ura3Δ5 CIT1-mCherry-hphMX4 PHO88-GFP(S65T)-KanMX6 mfb1Δ::LEU2*	This Study
WPY0023	*MAT**a** his3Δ1 leu2Δ0 met15Δ0 ura3Δ5 CIT1-mCherry-hphMX4 PHO88-GFP(S65T)-KanMX6 num1Δ::LEU2*	This Study
WPY0024	*MAT**a** his3Δ1 leu2Δ0 met15Δ0 ura3Δ5 CIT1-mCherry-hphMX4 PHO88-GFP(S65T)-KanMX6 num1Δ::LEU2 mfb1Δ::URA3*	This Study
WPY0015	*MAT**a** his3Δ1 leu2Δ0 met15Δ0 ura3Δ9 [pMito-roGFP1:URA3]*	This Study
WPY0016	*MAT**a** his3Δ1 leu2Δ0 met15Δ0 ura3Δ10 mfb1Δ::LEU3 [pMito-roGFP:URA3]*	This Study
WPY0017	*MAT**a** his3Δ1 leu2Δ0 met15Δ0 ura3Δ10 mmr1Δ::LEU3 [pMito-roGFP:URA3]*	This Study
WPY0018	*MAT**a** his3Δ1 leu2Δ0 met15Δ0 ura3Δ10 mmr1Δ::LEU3 mfb1Δ::KanMX6 [pMito-roGFP:URA3]*	This Study
JVY:66	*MAT**a** his3Δ1 leu2Δ0 met15Δ0 ura3Δ10 CIT1-mCherry-hphMX4 MFB1-GFP-KanMX6*	This Study
WPY0048	*MAT**a** his3Δ1 leu2Δ0 met15Δ0 ura3Δ10 CIT1-mCherry-hphMX4 MFB1-GFP-KanMX6 num1Δ::LEU3*	This Study
WPY0105	*MAT**a** his3Δ1 leu2Δ0 met15Δ0 ura3Δ10 CIT1-mCherry-hphMX4 NUM1-GFP-KanMX6*	This Study
WPY0106	*MAT**a** his3Δ1 leu2Δ0 met15Δ0 ura3Δ10 CIT1-mCherry-hphMX4 NUM1-GFP-KanMX6 mfb1Δ::URA3*	This Study
WPY0067-1	*MAT**a** his3Δ1 leu2Δ0 met15Δ0 ura3Δ17 TOM70-mCherry-hphMX4*	This Study
WPY067	*MAT**a** his3Δ1 leu2Δ0 met15Δ0 ura3Δ17 TOM70-mCherry-hphMX4 mfb1Δ::KanMX6*	This Study
WPY0068	*MAT**a** his3Δ1 leu2Δ0 met15Δ0 ura3Δ17 TOM70-mCherry-hphMX4 mmr1Δ::LEU2*	This Study
WPY0069	*MAT**a** his3Δ1 leu2Δ0 met15Δ0 ura3Δ17 TOM70-mCherry-hphMX4 mfb1Δ::KanMX6 mmr1Δ::LEU2*	This Study
SEY6210.1	*MAT**a** leu2-3,112 ura3-52 his3-Δ 200 trp1-Δ901 lys2-801 suc2-Δ9*	Robinson et al., 1988

ANDY201	*SEY6210.1 SEC61-GFP::TRP1 ist2Δ::HISMX6 scs2Δ::TRP1 scs22Δ::HISMX6 tcb1Δ::KANMX6 tcb2Δ::KANMX6 tcb3Δ::HISMX6*	Manford et al., 2012
WPY0031	*MAT**a** leu2-3,112 ura3-52 his3-Δ 200 trp1-Δ901 lys2-801 suc2-Δ9 CIT1-mCherry-hphMX4 PHO88-CFP-LEU2*	This Study
WPY0032	*SEY6210.1 SEC61-GFP::TRP1 ist2Δ::HISMX6 scs2Δ::TRP1 scs22Δ::HISMX6 tcb1Δ::KANMX6 tcb2Δ::KANMX6 tcb3Δ::HISMX6 CIT1-mCherry-hphMX4 PHO88-YFP-LEU2*	This Study

Supplementary Table 2: Primers used in this study

**Name**	**Sequence**
FW *MFB1* deletion	CCAACACAGTCTTCATACACTATTATTATTCATTTTATGGcggatccccgggttaattaa
RV *MFB1* deletion	CGTATAGTAGCTCTTTTTTTGTATCGATTTATAAAAATGCgaattcgagctcgtttaaac
FW *MFB1* deletion POM	CCAACACAGTCTTCATACACTATTATTATTCATTTTATGGacgctgcaggtcgacaaccc
RV *MFB1* deletion POM	CGTATAGTAGCTCTTTTTTTGTATCGATTTATAAAAATGCttaagggttctcgagagctc
FW *MMR1* deletion pOM	AAAAAAAAAAACACAACTAATAAACTAAACAACTAAAAAAacgctgcaggtcgacaaccc
RV *MMR1* deletion pOM	GTTTGTGTAAAATAAGTTAATTTAATTTGAAGTTGACGCTttaagggttctcgagagctc
FW *NUM1* deletion pOM	CTAATAGGACCACAGGGTTGAATAGAGACGAGTAAAGACGacgctgcaggtcgacaaccc
RV *NUM1* deletion pOM	TACTCATCGGTGGCAAACGTGTTTACAGGACTAAAAATCGttaagggttctcgagagctc
FW *PHO88* tag	AGAAGCTGAAAGAGCCGGTAACGCTGGTGTTAAGGCTGAAggtgacggtgctggttta
RV *PHO88* tag	TTCTATGGCGATGTAGGAAAATAGACACAATTCGTCTAGCatcgatgaattcgagctcg
FW linker-YFP pWJ1863	GGTGACGGTGCTGGTTTAATTAACAGTatgagtaaaggagaagaacttttcactgg
FW nested *PHO88*-tag pWJ1863	AGAAGCTGAAAGAGCCGGTAACGCTGGTGTTAAGGCTGAAggtgacggtgctggtttaattaac agt
RV *PHO88* tag	AAAACTAGGAAAAAAAAATACTTCGCTTTTGATCGAATCAatcgatgaattcgagctcg
FW *NUM1*-tag pFA6	ACATAGAGTACCACAAAGCCGATCATTTGGCAATTTACGAcggatccccgggttaattaa
RV *NUM1*-tag pFA6	CATATTTATTTCAGTCACAAAACAAAATTAAAGAATTCGTgggcagatgatgtcgagg
FW *MFB1*-tag pFA6	TGTAATCAAACGGCTTGACGCTAATACCGATTTTAATATAcggatccccgggttaattaa

RV *MFB1*-tag pFA6	CGTATAGTAGCTCTTTTTTTGTATCGATTTATAAAAATGCgaattcgagctcgtttaaac
FW *MFB1*-Citrine tag	TGTAATCAAACGGCTTGACGCTAATACCGATTTTAATATAggtgacggtgctggttta
RV *MFB1*-Citrine tag	CGTATAGTAGCTCTTTTTTTGTATCGATTTATAAAAATGCatcgatgaattcgagctcg
FW *CIT1* tag	AAAATACAAGGAGTTGGTAAAGAAAATCGAAAGTAAGAACcggtgacggtgctggttta
RV *CIT1* tag	TTTGAATAGTCGCATACCCTGAATCAAAAATCAAATTTTCCcatcgatgaattcgagctcg
FW *TOM70* tag	TCAAGAAACTTTAGCTAAATTACGCGAACAGGGTTTAATGcggtgacggtgctggttta
RV *TOM70* tag	TTGTCTTCTCCTAAAAGTTTTTAAGTTTATGTTTACTGTcatcgatgaattcgagctcg
